# A Systematic Review of Post-surgical Adverse Events Related to Hem-O-Lok Clips: A Commonly Used Device With Potential Complications

**DOI:** 10.7759/cureus.111805

**Published:** 2026-06-30

**Authors:** Badreldin Mohamed, Laurence Hunt, Abdalla Hassan, Sami Mohammed, Salma Babikir, Diya Mirghani, Dina Saleh, Hibatallah Bakhiet, Abdelrahman Maglad, Mohammed Zourob, Palanivelraju Gopalakrishnan

**Affiliations:** 1 Vascular Surgery/General Surgery, Cumberland Infirmary, Carlisle, GBR; 2 General Surgery, South Tyneside District Hospital, South Shields, GBR; 3 Trauma and Orthopaedics, North Cumbria Integrated Care NHS Foundation Trust, Carlisle, GBR; 4 Urology, Glangwili General Hospital, Carmarthen, GBR; 5 Urology, South Tyneside District Hospital, South Shields, GBR; 6 Surgery, Cumberland Infirmary, Carlisle, GBR; 7 General Surgery, Cumberland Infirmary, Carlisle, GBR; 8 General Surgery, Royal Victoria Infirmary, Newcastle Upon Tyne, GBR; 9 Ophthalmology, Red Sea University, Khartoum, SDN; 10 Emergency Department, Wexford General Hospital, Wexford, IRL; 11 Gastroenterology, Sunderland Royal Hospital, Sunderland, GBR; 12 General Surgery, University Hospital of North Tees, Stockton-on-Tees, GBR

**Keywords:** eroded hem-o-lock clips, hem-o-lock clips, hem-o-lock clips complication, migrating hem-o-lock clips, perforatinghem-o-lock clip

## Abstract

Since their introduction, Hem-o-lok clips (HOLCs) have become widely accepted and increasingly reliable devices among surgeons performing minimally invasive surgery due to their effectiveness in achieving vascular pedicle control and haemostasis. Although HOLCs are generally regarded as safe devices, various complications have been reported. This review aimed to identify and summarise complications occurring at any time following HOLC use, from their initial introduction in January 1999 through December 2025. This literature review was conducted in accordance with the PRISMA (Preferred Reporting Items for Systematic Reviews and Meta-Analyses) guidelines. A systematic search of PubMed (MEDLINE®), Google Scholar™, ResearchGate, and Embase® was performed using predefined search strings and Boolean operators to identify English-language articles reporting complications associated with HOLC use.

The most frequently reported postoperative complications associated with HOLCs occurred after prostatectomy, accounting for 66.23% (n = 51) of all reported cases. The second most common postoperative complication was associated with laparoscopic cholecystectomy, representing 14.28% (n = 11) of cases. The most common complication was clip migration to the urinary bladder with stone formation, accounting for 45.45% (n = 35), followed by urinary bladder neck contracture (14.29%; n = 11), clip migration to the ureter and erosion into the collecting system (14.29%; n = 11), erosion into the duodenum/resulting in duodenal ulcer formation (10.39%; n = 8), and common bile duct (CBD) obstruction (including Mirizzi syndrome or CBD stones) (3.89%; n = 3). Other less frequently reported complications, comprising 11.69% (n = 9), included clip migration to the urinary bladder without stone formation, clip migration to the rectum, erosion into the small intestine, transmural migration to the oesophagus, and anastomotic leak following prostatectomy.

Although HOLCs are generally considered safe devices, rare but clinically significant complications have been reported. A high index of clinical suspicion is essential for the early recognition and diagnosis of these complications. The management of HOLC-related complications varies according to the site and nature of the complication but most commonly involves endoscopic retrieval. Conservative management may be appropriate in selected asymptomatic patients.

## Introduction and background

Minimally invasive surgery has become the standard approach for most intra-abdominal procedures, including several complex reconstructive operations [1]. To facilitate these procedures, devices such as metal surgical clips, staplers, and Hem-o-lok clips (HOLCs) are used for vascular pedicle control, haemostasis, and to improve operative efficiency [1]. HOLCs, first introduced in 1999, are non-absorbable polymer clips featuring a locking mechanism and tactile feedback, which provide secure ligation, reduce the risk of tissue dislodgement, and produce minimal imaging artefacts [2].

HOLCs have become a trusted device among surgeons performing minimally invasive surgery because of their ability to control vascular pedicles, achieve haemostasis, and stabilise sutures [3]. Therefore, HOLCs are commonly used in robotic and laparoscopic urological operations to control the renal arteries and veins, to ligate the ureters, and to control bleeding points on the kidneys and prostate during radical cystectomy, prostatectomy, nephrectomy, or partial nephrectomy procedures. HOLCs are also used to control vascular pedicles during colectomies and the cystic duct and cystic artery during laparoscopic cholecystectomy [3,4]. Even though HOLCs are deemed to be relatively safe devices, rare complications have been reported, such as bleeding, erosion into nearby structures, external pressure on nearby structures, such as the ureter, urinary bladder, or duodenum, migration of the HOLCs, and stone formation in the common bile duct (CBD), urinary bladder, and ureters [3,4].

## Review

Materials and methods

Overview

This literature review was conducted per the PRISMA (Preferred Reporting Items for Systematic Reviews and Meta-Analyses), 2020 updated guidelines.

Eligibility Criteria

This literature review included case reports, case series, original articles, and literature reviews that reported HOLC-related complications in adult patients (>18 years), regardless of sex. Only studies authored by more than one author and published in English were included.

Information Sources

A systematic search was conducted using PubMed (MEDLINE®), Google Scholar™, ResearchGate, and Embase® databases. Predefined search strings and Boolean operators were used to identify English-language articles reporting complications associated with HOLC use. Grey literature and conference proceedings were also searched to minimise publication bias. The keywords used in our search were "Hem-o-lok clips, Hem-o-lok polymer clips, migrating clips, eroded clips, clip migration, surgical instruments, laparoscopic colorectal surgery, perforation, nephrectomy, ureteral calculi, prostatectomy, complication, bladder neck contracture, duodenal obstruction, and laparoscopic cholecystectomy."

The earliest article identified was published in July 2008. Among the included studies, 72.34% (n = 34) reported complications following urological procedures, accounting for 81.82% (n = 63) of all documented HOLC-related complications. The remaining 27.66% (n = 13) reported complications following gastrointestinal or hepato-pancreato-biliary (HPB) procedures, accounting for 18.18% (n = 14) of all reported complications. The search covered articles published between January 1999 (when HOLCs were first introduced) and December 2025. Additional relevant studies were identified through manual screening of the reference lists of the included articles. The final literature search was conducted on February 28, 2026. All retrieved records were exported to Microsoft Excel for screening and data management.

Selection Process

Eight investigators independently screened the titles and abstracts of the retrieved studies. Full-text articles were subsequently assessed for eligibility according to the predefined inclusion and exclusion criteria. Any disagreements or uncertainties among the investigators were resolved through discussion and consensus with the lead authors (BM and PG).

Data Extraction

Data extraction was independently performed by eight investigators using a standardised data collection sheet. Variables extracted from eligible studies included study design, sample size, publication date, index procedure, reported complications, clinical presentation, time to complication onset following the primary procedure, management of complications, study limitations, and study conclusions. Any discrepancies or uncertainties were resolved through discussion and consensus with the lead authors (BM and PG).

Study Outcomes Ethical Considerations

The primary outcome of this review was to evaluate reported complications occurring at any time following HOLC use. The secondary outcomes included assessment of the clinical presentation of HOLC-related complications, their timing relative to the index procedure, and associated management strategies. Informed written consent was not obtained from patients, as no identifiable patient data were used and no breach of confidentiality occurred. The authors declare no conflicts of interest and no external funding. Ethical approval was not required, as this study did not involve primary data collection or human participants.

Risk-of-Bias Assessment

The risk-of-bias assessment was conducted by six investigators (AH, BM, HB, LH, SB, and DM). The Risk-of-Bias Visualization tool (ROBVIS) was used to evaluate potential bias across the included studies in this systematic review. Any disagreements or uncertainties among the investigators were resolved through discussion and consensus with the lead authors (BM and PG).

Results

**Figure 1:**
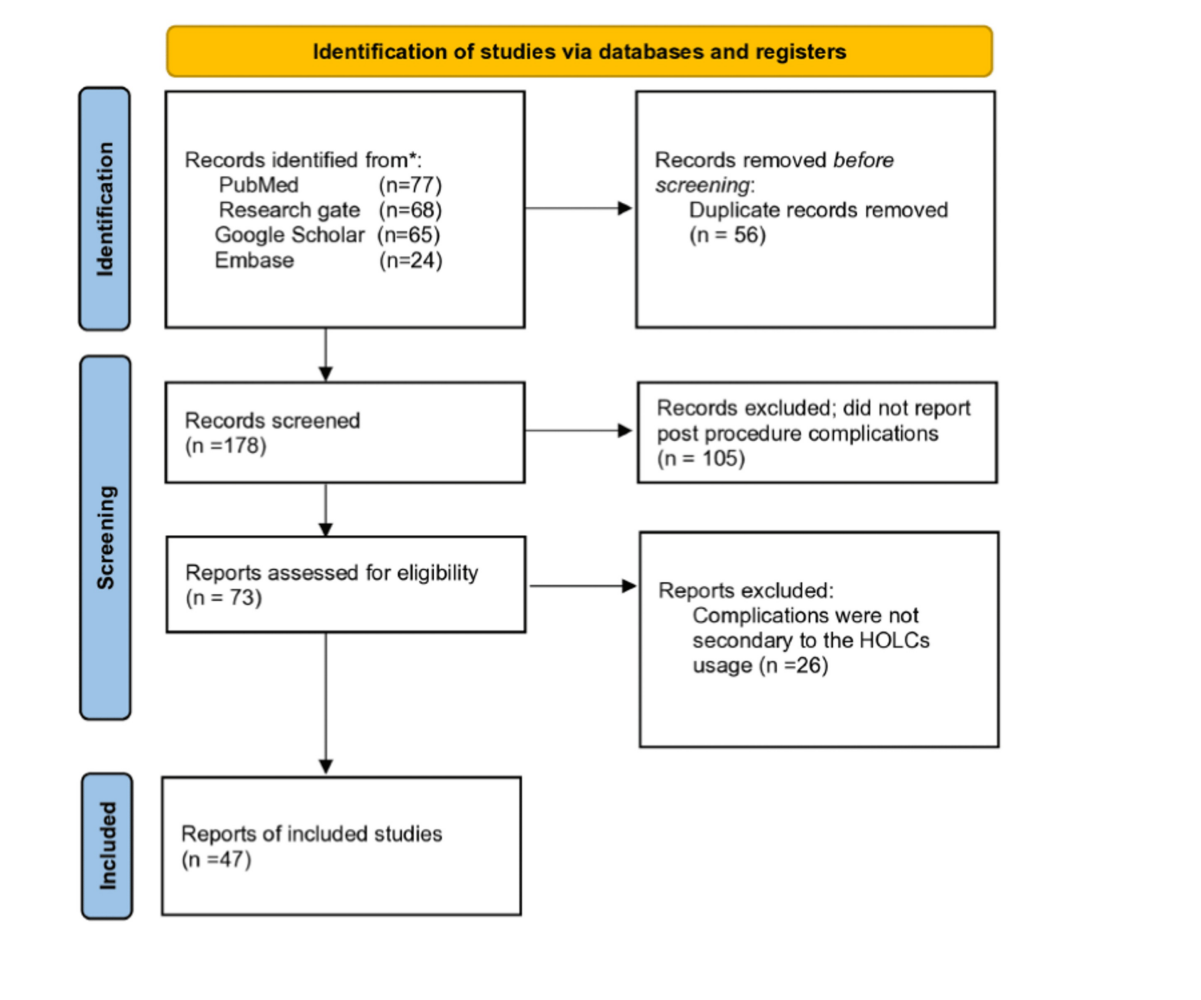
PRISMA flowchart depicting the study selection HOLCs: Hem-o-lok clips; PRISMA: Preferred Reporting Items for Systematic Reviews and Meta-Analyses

The initial search identified 234 articles. After removing duplicate articles (n = 56), 178 articles remained. Of these, 105 were excluded because they did not report post-procedure complications, and a further 26 articles were excluded because the reported complications were not secondary to the use of HOLCs. Therefore, 47 articles were deemed eligible for inclusion in this systematic review (Figure 1).

The first article was published in July 2008. Of these publications, 72.34% (n = 34) reported post-urological procedures, and these accounted for 81.82% (n = 63) of the recorded complications following HOLC use. The remaining 27.66% (n = 13) of the articles reported post-gastrointestinal or HBP procedures, and they accounted for 18.18% (n = 14) of the complications following HOLC use. The screening process and the characteristics of the included studies are shown in Figure 1.

The most commonly reported postoperative complications occurred after prostatectomy (both robot-assisted and laparoscopic prostatectomy) and accounted for 66.23% (n = 51) of all reported complications. Complications following laparoscopic cholecystectomy were the second most commonly reported, accounting for 14.28% (n = 11) of all reported complications associated with HOLC use (Table 1).

**Table 1:** Review of the reported complications vs. procedures

Procedure	N	%
Robot-assisted radical prostatectomy	45	58.44%
Laparoscopic radical prostatectomy	6	7.79%
Partial nephrectomy	7	9.09%
Laparoscopic orthotopic neobladder cystectomy	1	1.30%
Laparoscopic pyelolithotomy	2	2.60%
Bilateral robotic pyeloplasty	1	1.30%
Retroperitoneoscopic left pelvilithotomy	1	1.30%
Laparoscopic cholecystectomy	11	14.28%
Colectomy	2	2.60%
Minimally invasive oesophagectomy	1	1.30%

The clinical presentation of the reported complications varied depending on the location of the HOLCs. The most commonly reported presentation was lower urinary tract symptoms, such as dysuria, haematuria, and suprapubic pain, which were reported in 54.55% (n = 42) of patients, all of whom had undergone prostatectomy. Upper urinary tract symptoms, such as renal colic, were reported in 23.38% (n = 18) of the patients due to HOLCs becoming impacted in the ureter, resulting in obstructive uropathy with or without secondary infection and stone formation. This presentation was mainly reported after renal procedures, such as partial nephrectomy, pyeloplasty, and pyelolithotomy. Three patients (3.89%) presented with obstructive jaundice. Two patients (2.60%) presented with complicated CBD stones. One patient (1.30%) had obstructive jaundice and biliary sepsis, whereas in the other patient (1.30%), the clip exerted external pressure on the CBD, resulting in obstructive jaundice and Mirizzi syndrome.

Two patients (2.60%) presented with gastrointestinal bleeding due to HOLCs eroding into the duodenal bulb. In another six patients (7.79%), the HOLCs had eroded into the duodenum. Of these, three patients (3.89%; n = 3) presented with vague abdominal symptoms, whereas the remaining three patients were asymptomatic and were diagnosed during esophagogastroduodenoscopy (EGD) performed for other reasons (Tables 2, 3, 4).

**Table 2:** Presentation of HOLC-related complications HOLCs: Hem-o-lok clips

Presentation	N	%
Lower urinary tract symptoms	42	54.55%
Obstructive uropathy	13	16.88%
Renal colic	5	6.49%
Abdominal pain	3	3.89%
Asymptomatic	3	3.89%
Urine retention	2	2.60%
Small bowel obstruction	2	2.60%
Obstructive jaundice	2	2.60%
Malena	2	2.60%
Acute cholangitis	1	1.30%
Dysphagia	1	1.30%
Loose motions	1	1.30%

**Table 3:** Presentation of HOLC-related complications from the initial operation HOLCs: Hem-o-lok clips

Time from operation	N	%
2 weeks	1	1.30%
2 weeks - 3 months	15	19.48%
4 - 6 months	13	16.88%
7 - 12 months	12	15.59%
13 - 24 months	25	32.47%
25 - 36 months	1	1.30%
37 - 60 months	5	6.49%
More than 60 months	5	6.49%

**Table 4:** HOLC-related complications HOLCs: Hem-o-lok clips; CBD: common bile duct; SBO: small bowel obstruction

Complications	N	%
Clip migration to the bladder with stone formation	35	45.45%
Bladder neck contracture	11	14.29%
Migration to ureter and erosion to collecting system	11	14.29%
Erosion to the duodenum/ duodenal ulcer	8	10.39%
CBD obstruction (Mirizzi syndrome/CBD stone)	3	3.89%
Clip migration to the bladder	3	3.89%
Clip migration to the rectum	2	2.60%
Erosion to small intestine/SBO	2	2.60%
Transmural migration to the oesophagus	1	1.30%
Anastomotic leak post prostatectomy	1	1.30%

Of the reported patients, two (2.60%, n = 2) presented with small bowel obstruction. One patient underwent an elective laparoscopic reversal of Hartmann’s procedure following an elective laparoscopic sigmoid colectomy complicated by an anastomotic leak, whereas the second patient also underwent an elective laparoscopic reversal of Hartmann’s procedure for perforated sigmoid diverticulitis. In both patients, HOLCs were used to control the inferior mesenteric artery pedicle. Ultimately, the clips exerted pressure on the adjacent small intestine serosa, resulting in bowel stricture and erosion into the small intestine. Both patients underwent small bowel resection and anastomosis. One patient (1.30%, n = 1) underwent a minimally invasive esophagectomy, which was complicated by transmural migration of the HOLC from the azygos vein stump into the gastric conduit, causing worsening dysphagia.

The subsequent gastroscopy revealed a normal gastric conduit with two HOLCs containing the azygos vein stump within its lumen at the level of the oesophago-gastric anastomosis, where they exerted an obstructive effect. The clips were successfully removed by EGD. In 10.39% of the patients (n = 8), HOLCs eroded into the duodenum. All clips were located in the duodenal bulb except in one patient (1.30%), in whom the clip eroded into the third part of the duodenum (Tables 2, 3, 4). A summary of the management of post-HOLC complications is presented in Table 5.

**Table 5:** Management of HOLC-related complications HOLCs: Hem-o-lok clip; BNC: bladder neck contracture; EGD: esophagogastroduodenoscopy; ERCP: endoscopic retrograde cholangiopancreatography

Management	N	%
Lithotripsy and HOLC extraction	31	40.26%
HOLC extraction by cystoscopy	20	25.97%
Post-BNC dilation	7	9.09%
HOLC extraction by EGD	5	6.49%
Conservative management	4	5.19%
Small bowel resection and anastomosis	2	2.60%
HOLC extraction by ERCP	2	2.60%
Colonoscopy extraction	2	2.60%
Cold bladder knife incision	1	1.30%
Percutaneous nephrostomy	1	1.30%
Spontaneous release	1	1.30%
Bladder neck resection	1	1.30%

**Figure 2:**
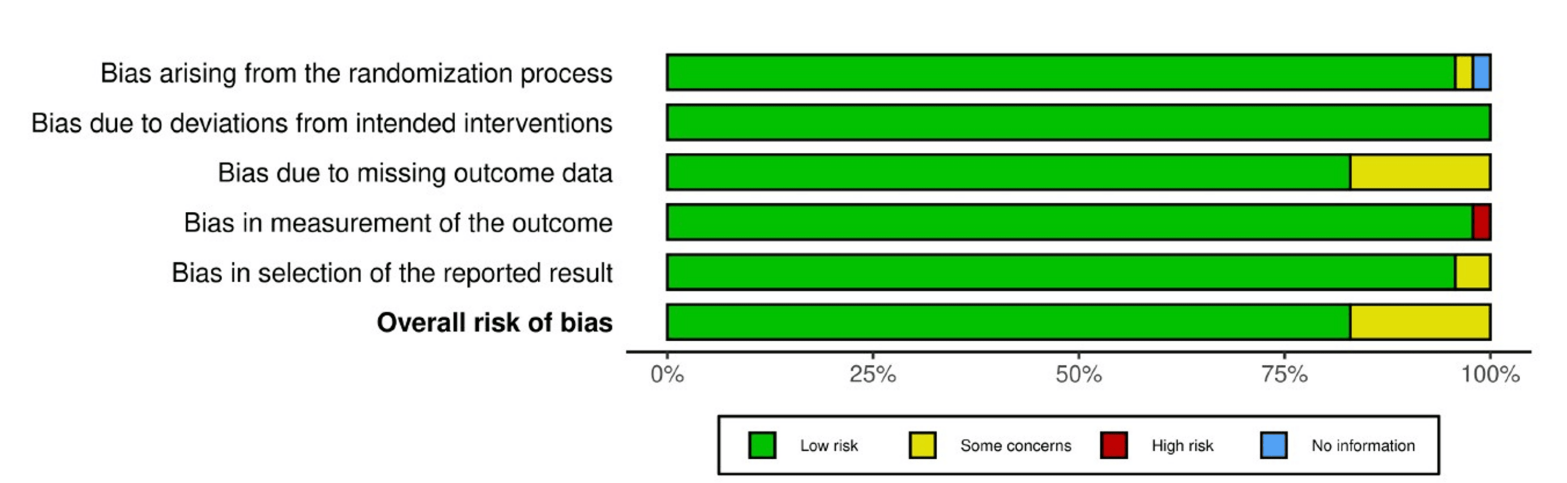
ROBVIS instrument assessment for non-randomised, non-comparative studies ROBVIS: Risk of Bias Visualization tool

ROBVIS was used to assess the risk of bias in our systematic review. The assessment identified some concerns regarding bias due to missing data, mainly patient age, post-procedure outcome, and post-procedure follow-up information (Figure 2).

Discussion

HOLCs have been widely used in different gastrointestinal and urological surgeries since 1999, but their use is not free from complications [5]. Most reported HOLC complications are caused by clip migration, improper clip locking, or mechanical pressure effects on nearby structures, such as a rectal submucosal lesion following prostate surgery, dysphagia following esophagectomy, or obstructive uropathy. This systematic review aimed to assess the complications associated with HOLC use in the surgical field, and the majority of the included studies were case reports and case series [6].

The mechanism of HOLC migration is unknown. However, Lee et al. proposed a mechanism following urological procedures, suggesting that an unintentional violation of the collecting system during partial nephrectomy or excessive pressure applied to clips during firing may provoke clip migration through erosion. Additionally, poor healing due to conditions such as diabetes mellitus or chronic kidney disease may contribute to HOLCs migration [7].

Yu et al. describe three different types of HOLCs migration: (1) which typically occurs between two and eight months, and patients present with lower urinary tract symptoms; this has been reported in 54.55% (n = 42) of the assessed patients; (2) in which patients form stones and present with haematuria; this has been reported in 45.45% (n = 35) of the reported cases; and (3) which occurs in the weeks after the operation, where patients spontaneously expel the clip, and this has been reported in 1.30% (n = 1) of the reported patients [8]. HOLCs are likely to pass spontaneously if they migrate to the lower urinary tract. In the upper urinary tract, however, because of its anatomy and the shape of the HOLCs, they are likely to cause obstructive uropathy and subsequently hydronephrosis and stone formation, and this was reported in 16.88% (n = 13) of the assessed patients [9].

As HOLCs are radiolucent, it is difficult to see them in the urinary tract until stone formation, and therefore, a high clinical suspicion is required for prompt diagnosis [10]. Matsushita et al. reported that HOLCs' density is 223 Hounsfield units (HU), which can be visualised by ex vivo CT-based attenuation measurements [10]. The recommended approach to remove migrated HOLCs is an endoscopic approach, as the clip might be tightly attached to renal parenchyma with or without lithotripsy [11]. Lithotripsy and endoscopic HOLC extraction were reported in 40.26% (n = 31), and HOLC extraction by cystoscopy alone was reported in 25.97% (n = 20) of the assessed cases. Blumenthal et al. reported the first case of HOLC migration to the urinary bladder post-prostatectomy, which ultimately led to stone formation and urinary bladder neck contracture (BNC) [12]. The authors recommended avoiding the use of HOLCs to control bleeding near the urethro-vesical anastomosis or bladder neck and removing any loose clips to prevent migration and inflammatory processes, which ultimately lead to BNC. This has been reported in 14.29% (n = 11) of the assessed complications [12,13].

Multiple different approaches to the management of BNC after HOLC removal were reported. For example, one method is potassium-titanyl-phosphate (KTP) laser vaporisation of the stricture followed by self-catheterisation to prevent stricture recurrence [12,13]. Cormio et al. performed a cold-knife urethral incision and wide bladder neck resection in 1.30% (n = 1) of the assessed patients, but this was associated with incontinence [14]. It was unclear whether the patient developed incontinence because of excessive resection or because of fibrosis associated with the inflammatory process after BNC, which may have already weakened the sphincter before the intervention [14]. Alternatively, clip removal and urethral dilatation without the need for catheterisation were successful in seven cases (9.09%) [15].

In biliary procedures, HOLC migration has been explained by different mechanisms: (1) ineffective cystic duct closure, in which bile leak occurs from a partially patent cystic duct; (2) direct erosion or invasion of the HOLCs applied to the cystic duct into the duodenal wall, either because of anatomical proximity, or if the duodenum has been pulled next to the clipped cystic duct or artery stump due to inflammatory processes from a difficult Calot’s triangle dissection, acute cholecystitis, or pancreatitis, ultimately leading to a fistula between the duodenum and biliary tree; (3) an already weakened duodenal wall from a pre-existing ulcer can be pulled into the gallbladder fossa by inflammatory processes, bringing the duodenum into contact with the clips, which may erode through the ulcerated duodenal wall; in our review, 7.79% (n = 8) eroded into the duodenum, but these cases did not report possible predisposing factors for duodenal erosion; (4) a growing mass in the upper gastrointestinal tract distorting the anatomy, causing the duodenal wall to come into contact with post-cholecystectomy HOLCs; (5) the use of four or more HOLCs on the cystic duct may exert mechanical pressure on the CBD, which may eventually erode the CBD and serve as a nidus for CBD stone formation; this has been reported in 2.60% (n = 2) of the reviewed patients; (6) mechanical pressure of the liver on the cystic duct stump may push the inverted clip into the CBD, which may again act as a nidus for ductal stones or exert external pressure on the CBD, as reported in 1.30% (n = 1) of the reviewed patients [16,17].

Migrated clips in the biliary tree can have different presentations, such as CBD stones and obstructive jaundice, as reported in two patients (2.60%), cholangitis as reported in one patient (1.30%), shooting off clip emboli, and ulcer or fistula formation typically with the duodenum [17]. A migrated clip to the duodenal bulb can present with vague abdominal pain, as reported in three patients (3.89%), macroscopic or microscopic gastrointestinal blood loss, as has been reported in two patients (2.60%), or iron deficiency anemia. The endoscopic findings are mucosal erosion, which can progress to an ulcer, and if the mucosa is intact over the clip, it can mimic submucosal tumors, carcinoid tumors, polyps, or cystic lesions [18].

Avoidance of HOLC-related complications can be achieved by meticulous dissection of Calot’s triangle, ultrasonic dissection, appropriate placement of the clips, and avoiding a high number of clips at the cystic duct. The ulcerated clip in the duodenal bulb can be retrieved by EGD if it has already entirely eroded through the duodenal wall, as reported in 6.49% (n = 5) of the reviewed patients, or it can be managed medically with proton pump inhibitors if it is a superficial ulcer or if the clip has only partially eroded into the duodenal wall, as reported in one patient (1.30%). If the patient is asymptomatic, they can be managed conservatively, as reported in three patients (3.89%) [18,19].

Limitations

This systematic review inherently depends on the accuracy and completeness of the included studies, which represents one of its key limitations. It also has a retrospective design, making it susceptible to selection bias. Although the included articles are multicentric in nature, the overall number of reported cases remains relatively small, and they describe a wide range of complications across different specialties and procedures, which may have influenced the overall findings. This is primarily due to the fact that an already small study population was further subdivided into even smaller subgroups. An additional limitation is the inclusion of data derived from heterogeneous case reports, which introduces further variability in the reported outcomes.

## Conclusions

Even though HOLCs are deemed to be relatively safe devices, rare complications have still been reported. A high index of clinical suspicion is essential for early diagnosis of such complications and prompt intervention. The management of HOLC-related complications varies according to their location, but they are most commonly treated by endoscopic retrieval and, in rare cases, may be managed conservatively in asymptomatic patients. Preventive strategies include meticulous dissection and careful removal of misplaced or migrated clips. Surgeons should be adequately trained in the correct and safe use of HOLC devices, with particular attention to proper transfixation techniques.
